# Effect of co-vaccination of cattle with RB51 and BCG on vaccine-specific CD4^+^ T cell responses

**DOI:** 10.3389/fimmu.2025.1664398

**Published:** 2025-08-27

**Authors:** Haley M. Sterle, Ellie J. Putz, Mitchell V. Palmer, Steven C. Olsen, Paola M. Boggiatto

**Affiliations:** ^1^ National Animal Disease Center, Agricultural Research Service (USDA), Ames, IA, United States; ^2^ Oak Ridge Institute for Science and Education, Oak Ridge, TN, United States

**Keywords:** BCG, RB51, CD4, cattle, T helper 1 (IFN-γ)

## Abstract

**Introduction:**

*Brucella abortus* and *Mycobacterium bovis*, the causative agents of bovine brucellosis and tuberculosis respectively, are zoonotic bacterial pathogens that both contribute to major economic losses in the cattle industry and pose a human health risk worldwide. Co-infections of cattle with *B. abortus* and *M. bovis* have been identified in various developing countries, necessitating the development of an efficacious strategy for controlling both important zoonotic diseases even in the event of co-infection. *Brucella abortus* strain RB51, a live attenuated vaccine for bovine brucellosis that is currently used in the US, is highly effective at preventing reproductive failure due to brucellosis in cattle. Bacillus Calmette-Guérin (BCG) is a live attenuated vaccine strain of *M. bovis* that provides protection against bovine tuberculosis in cattle but is not currently licensed for use in the US.

**Methods:**

The study presented here compares functional Th1 responses of RB51 + BCG vaccinated cattle to responses of RB51-only and BCG-only vaccinated cattle to evaluate the feasibility of a combined vaccination strategy for controlling both bovine brucellosis and tuberculosis.

**Results:**

This work identified that peripheral blood mononuclear cells (PBMC) from RB51 vaccinates proliferate not only in response to stimulation with killed RB51 but also in response to mycobacterial antigen PPDb. Combination vaccinates show significantly more CD4^+^ T cell proliferation than single BCG vaccinates when stimulated with PPDb, while no differences were observed between RB51 and combination vaccinates stimulated with RB51.

**Discussion/conclusion:**

Significantly enhanced BCG-specific Th1 responses in combination vaccinates compared to BCG-only vaccinates suggest that combining vaccinations for *B. abortus* and *M. bovis* may alter the host CD4^+^ T cell response.

## 1 Introduction

*Brucella abortus* is a zoonotic pathogen that causes reproductive failure and abortion in cattle and other reservoir species (1), and is the etiologic agent of bovine brucellosis. In the United States, bovine brucellosis is endemic in elk and bison in the Greater Yellowstone Area, which encompasses parts of Idaho, Montana, and Wyoming. Domestic cattle herds in these states are therefore at risk of contracting brucellosis from infected elk or bison (2). Transmission of brucellosis most often occurs via the mucosa, either orally or through aerosols from contact with tissue from the birth or abortion of an infected fetus (1). As such, the most important objective to reduce transmission in the field is to prevent fetal infection and abortions. In addition to serologic testing and removal of infected animals from herds, current brucellosis control strategies in the United States focus on the calfhood vaccination of heifers with *B. abortus* strain RB51 (RB51) between 4 and 12 months of age (1, 3). RB51 is highly effective at preventing abortion in cattle (4, 5) and, importantly, does not interfere with serological diagnostic testing for brucellosis infection (6). Therefore, RB51 functions as a DIVA vaccine (Differentiation of Infected from Vaccinated Animals).

Adaptive cellular immunity, specifically a T helper type 1 (Th1) response, is essential for protection against brucellosis (7). Studies have shown that the production of interferon-γ (IFN-γ) by *Brucella*-specific CD4^+^ T cells is important for macrophage activation and clearance of the bacteria (8–11). Murine studies indicate that while CD8^+^ T cells are dispensable for protection against *Brucella*, CD4^+^ T cells are required (11, 12). Though a strong humoral response is induced following exposure to *Brucella*, antibodies do not contribute significantly to protection against infection or prevention of abortion (11, 13). Our own previous work has demonstrated that vaccination of cattle with RB51 results in proliferative and IFN-γ responses in peripheral blood mononuclear cells (PBMCs) from vaccinated animals, and these responses are associated with protection against *B. abortus* challenge (14, 15). However, questions remain regarding the specific cell types responsible for antigen-specific responses. Dorneles et al. addressed this knowledge gap by evaluating peripheral lymphocyte responses to RB51 vaccination using an *in vitro* recall response assay (16). In that study, surface markers and cytokine production of PBMCs were evaluated using two-, three-, or four-color flow cytometry panels. The percentage of *Brucella*-specific CD4^+^ T cells peaked at 4 weeks post-vaccination (PV) using proliferation to *Brucella* antigens as a measure of vaccine specificity, and CD4^+^ T cells produced IFN-γ in response to antigen stimulation. Among other observations, their study concluded that RB51 vaccination induces *Brucella*-specific, Th1 polarized immune responses in cattle.

Another zoonotic intracellular pathogen, *Mycobacterium bovis*, is the causative agent of bovine tuberculosis (bTB). Transmission occurs via inhalation of aerosolized bacteria from a reservoir host with an active infection (17). Following infection, bacteria mainly reside in macrophages and cause granulomatous lesions of the lung and pulmonary lymph nodes (18). The lack of a highly efficacious vaccine for bTB, coupled with persistent infections in wildlife reservoirs, prevents eradication of the disease in domestic cattle herds (19). Similarly to *Brucella*, a Th1 immune response is instrumental in controlling mycobacterial infections. *M. bovis*-specific CD4^+^ T cells producing IFN-γ enhance the bactericidal activity of macrophages to encourage the clearance of *M. bovis* (20, 21). However, increased IFN-γ production does not directly correlate to protection against bTB (22, 23). Humoral responses to *M. bovis* tend to correlate positively with bacterial persistence and pathology, indicating that high antibody titers are not sufficient for protection (24, 25).

Bacillus Calmette-Guerin (BCG) is a live attenuated strain of *M. bovis* that was developed for human vaccination against *Mycobacterium tuberculosis* infections in the early 20^th^ century. Though BCG is not used in the United States, infants are still vaccinated in developing countries where tuberculosis is prevalent (26). BCG is also known to be protective against bTB in cattle and wildlife reservoirs, reducing lesion severity in cattle and various wildlife species under controlled experimental conditions (22, 23, 27–30). However, vaccination with BCG can interfere with currently approved bTB diagnostic testing methods for cattle (31) and is not approved for use in the United States. Despite efforts to develop other efficacious DIVA vaccines for bTB, BCG remains a front runner in the effort to control the disease (19).

Though uncommon in the United States, co-infections with *Brucella abortus* and *Mycobacterium bovis* in domestic cattle herds and wildlife have been reported in other countries including Canada, South Korea, Burkina Faso, and Nigeria (32–36). Globally, there is also considerable geographic overlap between high-risk areas for brucellosis and bTB (37–41). Reducing the prevalence of both pathogens in livestock is important to minimize economic losses and prevent zoonoses in farm workers. Development of a safe, efficacious, combined vaccine for bovine brucellosis and tuberculosis may be an efficient option to address co-infections in cattle herds worldwide.

In addition to characterizing the peripheral CD4^+^ T cell response to RB51 vaccination in cattle, we sought to determine whether co-administration of two live attenuated bacterial vaccines, RB51 and BCG, influenced the CD4^+^ T cell response to either vaccine. Our data provide additional foundational knowledge on the bovine immune response to RB51 vaccination and suggest a vaccination strategy to enhance BCG-specific immune responses.

## 2 Materials and methods

### 2.1 Vaccination

Two studies using twenty-four yearling dairy-beef crossbred heifers and twenty-four four-month-old Holstein steer calves were conducted at the National Animal Disease Center (NADC) in Ames, Iowa. Crossbred heifers were randomly assigned into one of three vaccine groups (n = 8/group) and housed outdoors. RB51 vaccinates were vaccinated subcutaneously with 2 mL of 1x10^10^ colony forming units (CFU) of RB51 (Colorado Serum Company, Denver, CO). BCG vaccinates were vaccinated subcutaneously with 1 mL of 2.4x10^5^ CFU of BCG Danish strain 1331. Combo vaccinates were vaccinated subcutaneously with RB51 and BCG administered together in the same syringe. Holstein steers were randomly assigned to one of three treatment groups including control animals (n = 8), BCG vaccinates (n = 7), and RB51 + BCG combo vaccinates (n = 8). Vaccination was conducted as previously described for the study in dairy-beef crossbred heifers, using 1x10^10^ CFU RB51 and 1.36x10^6^ CFU BCG. Dose variability of BCG between the two studies was within standard accepted range (42, 43).

Standard veterinary protocols were implemented to maintain health and wellbeing of all animals. All animal procedures were approved prior to the studies by the NADC Animal Care and Use Committee.

### 2.2 Peripheral blood mononuclear cell isolation

Whole blood was collected from crossbred heifers at the time of vaccination and at four-week intervals until 24 weeks PV to assess peripheral immune responses. Blood was collected via jugular venipuncture in acid citrate dextrose (ACD) and PBMCs were then isolated as previously described (44). Live cell count was determined using the Muse^®^ Count and Viability Kit on the Guava^®^ Muse^®^ Cell Analyzer (Cytek, Fremont, CA) and cell suspensions were adjusted to a final concentration of 1x10^7^ cells per mL of complete 1640 RPMI (cRPMI) media containing 20% heat-inactivated FBS, 1% HEPES, 1% non-essential amino acids, 1% essential amino acids, 1% sodium pyruvate, 100 U/ml penicillin, 100 µg/ml streptomycin, 2nM glutamine, and 50 µM 2-beta mercaptoethanol. In the Holstein steer study, whole blood was collected at the time of vaccination and at 12- and 24- weeks PV and PBMCs isolated in the same manner.

### 2.3 Labeling PBMCs for proliferation assay

PBMCs from all animals were labeled with the CellTrace™ Violet (CTV) proliferation kit (Cat. No. C34557, Thermo Fisher Scientific, Waltham, MA) according to the manufacturer’s recommendations to track *in vitro* proliferation. Briefly, CTV dye was reconstituted in 20 µL dimethyl sulfoxide (DMSO) provided by the manufacturer, suspended in 780 µL PBS, and further diluted 1:10 in sterile Dulbecco’s phosphate buffered saline (DPBS). PBMCs were washed in DPBS and centrifuged at 300x g for 10 minutes (min) at room temperature (RT). Supernatants were discarded and cell pellet was resuspended in diluted CTV, then vortexed and incubated for 20 min at RT with occasional vortexing. Cells were then washed in DPBS and again centrifuged at 300x g for 10 min at RT. Supernatants were discarded, and PBMC were resuspended to a final concentration of 1x10^7^ cells per mL in cRPMI.

### 2.4 *In vitro* recall response assay

To assess *in vitro* recall responses, 100 µL of CTV-labeled cRPMI PBMC suspensions (1x10^6^ cells) were plated per well in 96 well flat bottom plates and incubated in duplicate wells without stimulation (media only) or with γ-irradiated RB51 (10^7^ CFU/well), purified protein derivative of *M. bovis* (PPDb) (30 μg/well), or Concanavalin A (ConA) (0.5 μg/well). Plates were incubated for 7 days at 37 °C with 5% CO_2_. Sixteen hours prior to beginning cell staining procedure on day 7, PBMCs were treated with eBioscience™ protein transport inhibitor (Cat. No. 00-4980-93, Thermo Fisher Scientific) to assess intracellular cytokine production.

### 2.5 Surface marker and intracellular cytokine staining

PBMCs were harvested on day 7 and washed twice in DPBS at 300x g for 5 min at RT. PBMCs were then incubated with eBioscience eFluor™ 780 fixable viability dye (Cat. No. 65-0865-14, Thermo Fisher Scientific) for 20 min at 4°C and subsequently washed via centrifugation under the same conditions as previously described, once with DPBS and once with FACS buffer (PBS + 0.5% fetal bovine serum (FBS)). Cells were then incubated with FITC labeled anti-bovine CD4 (clone CC8, Cat. No. MCA1653F, BioRad, Hercules, CA) antibody for 15 min at RT. Following incubation and two washes in FACS buffer, cells were fixed and permeabilized using the BD Cytofix/Cytoperm kit (Cat. No. 554714, BD Biosciences, Franklin Lakes, NJ) according to manufacturer’s recommendations. Intracellular staining was then carried out by incubating cells with PE labeled anti-bovine IFN-γ antibody (clone CC302, Cat. No. MCA1783PE, BioRad) for 30 min at RT. Cells were washed once with 1X wash/perm buffer and once with FACS buffer. Cells were then resuspended in 200 µL FACS buffer and concurrently analyzed for proliferation, intracellular cytokine production, and surface markers using a BD FACSymphony A5 flow cytometer (BD Biosciences). Data were analyzed using FlowJo software (version 10.8).

### 2.6 Statistical analysis

The vaccination study conducted with crossbred heifers was designed to follow vaccine-induced T cell responses over 24 weeks, comparing functional and phenotypic results back to Day 0 pre-vaccination time point, which was used as the control. Internal assay controls to evaluate the effect of environmental factors were included at each time point with unstimulated and ConA stimulated PBMCs. The study conducted with Holstein steers was designed to include challenge with *M. bovis* after 24-week evaluation of vaccine-induced T cell responses, therefore, unvaccinated control animals were included as a treatment group. The internal assay controls used in the dairy-beef crossbred heifer study were also implemented in the Holstein steer study.

All data were analyzed using a simple auto regressive model (AR1) in R (4.2.0). Time point (weeks post-vaccination), stimulation condition or vaccination group, and the interaction between time point and stimulation condition or vaccination group were set as fixed effects for all data. Pairwise comparisons of Least Squares Means were conducted to determine significant differences between specific contrasts of interest. Data are presented as mean ± SEM with statistical differences identified when P-value ≤ 0.05. Data presented here is open source and available at: Ag Data Commons 10.15482/USDA.ADC/26871160 and was analyzed as detailed below.

## 3 Results

### 3.1 RB51-specific CD4^+^ T cell recall response following RB51 vaccination of crossbred heifers

**Figure 1:**
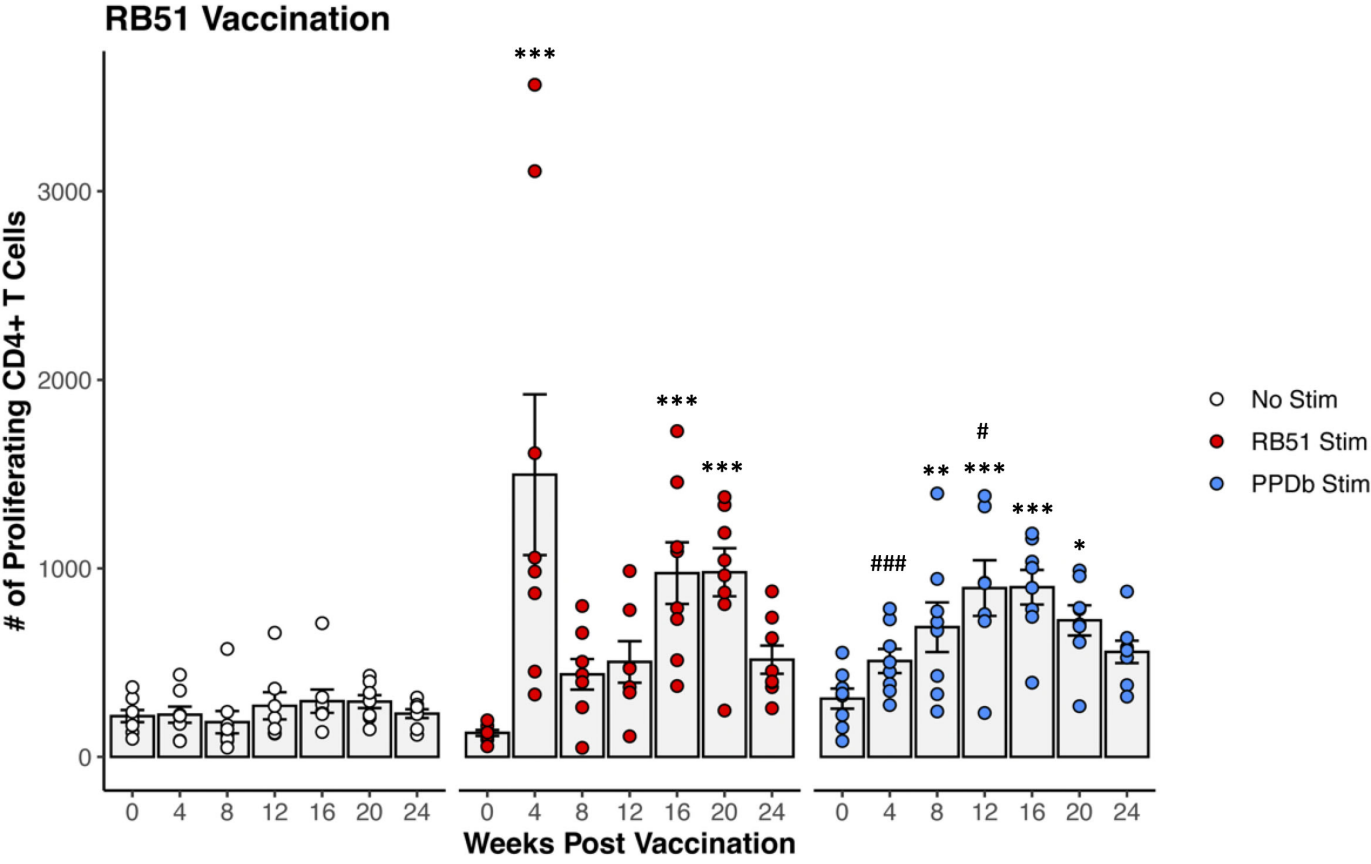
Proliferation of CD4^+^ T cells from RB51 vaccinated dairy-beef heifers in response to *in vitro* RB51 and PPDb stimulation. Mean numbers (gray bars) of CD4^+^ T cells proliferating in response to media only (no stimulation, white circles, n = 8), γ-irradiated RB51 (red circles, n = 8), and PPDb (blue circles, n = 8). *, RB51 or PPDb stimulated CD4^+^ T cells compared to unstimulated CD4^+^ T cells at respective time points. #, PPDb stimulated CD4^+^ T cells compared to RB51 stimulated CD4^+^ T cells at respective time points. AR1, circles represent individual animal cell counts and gray bars represent means ± SEM. *, #p ≤ 0.05; **p ≤ 0.01; ***, ###p ≤ 0.001.

PBMCs were isolated from RB51 vaccinated crossbred heifers, stimulated *in vitro* under various conditions, and analyzed via flow cytometry to identify RB51-specific CD4^+^ T cells using proliferation (**Supplementary Figure S1**). When PBMCs were left unstimulated (open circles), there were no changes in the number of proliferating CD4^+^ T cells (**Figure 1**). As expected, stimulation of PBMCs from RB51 vaccinated heifers with γ-irradiated RB51 (red circles) resulted in a significant increase in numbers of proliferating CD4^+^ T cells as compared to unstimulated negative controls (p < 0.001) at 4-, 16-, and 20-weeks PV. Peak proliferative responses were observed at 4-weeks PV, followed by a drop at 8-weeks PV, and a subsequent increase in the number of proliferating CD4^+^ T cells at 16-weeks PV. These data are consistent with previously reported results (16, 45), demonstrating significant RB51-specific CD4^+^ T cell responses in the peripheral blood of RB51 vaccinated cattle.

### 3.2 Mycobacterium-specific CD4^+^ T cell recall response following RB51 vaccination of crossbred heifers

When PBMCs from RB51 vaccinated crossbred heifers were stimulated with the unrelated bacterial antigen PPDb, we observed measurable proliferative responses. PPDb stimulation (blue circles) of PBMCs from RB51 vaccinated heifers resulted in increased numbers of proliferating CD4^+^ T cells when compared to unstimulated PBMCs at 8-, 12-, 16-, and 20-weeks PV (p <0.004, p <0.001, p < 0.001, p < 0.02, respectively) (**Figure 1**). The magnitude of the PPDb-driven CD4^+^ proliferative response was similar to that of the RB51-specific proliferative response at 8-, 16-, and 20-weeks PV (p > 0.1 for all), and was greater (p = 0.0445) at 12-weeks PV. However, the kinetics of the response differed from that observed after RB51 antigen stimulation, with peak proliferative responses observed at 12- and 16-weeks PV. Our data suggest that RB51 vaccination resulted in the generation of PPDb-responsive CD4^+^ T cells.

### 3.3 Proliferative response of CD4^+^ T cells following combo vaccination of crossbred heifers with RB51 and BCG

**Figure 2:**
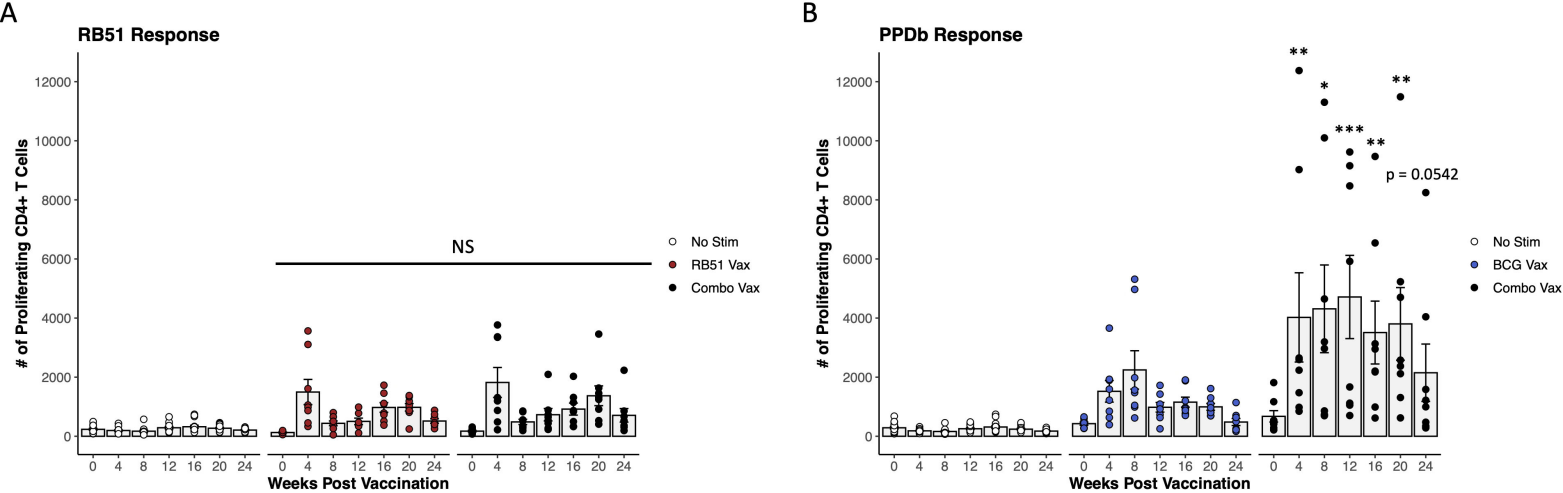
Proliferative CD4^+^ T cell responses in combo vaccinated dairy-beef heifers compared to RB51 or BCG vaccinates. **(A)** Mean numbers of RB51-specific CD4^+^ T cells (gray bars) in combo vaccinated heifers (black circles, n = 8) compared to RB51 vaccinated heifers (red circles, n = 8). Proliferation in unstimulated (white circles, n = 16) CD4^+^ T cells from both RB51 and combo vaccinated heifers was used as a control. **(B)** Mean numbers (gray bars) of BCG-specific CD4^+^ T cells in combo vaccinated heifers (black circles, n = 8) compared to BCG vaccinated heifers (blue circles, n = 8). Proliferation in unstimulated (white circles, n = 16) CD4^+^ T cells from both BCG and combo vaccinated heifers was used as a control. *, proliferating CD4^+^ T cells from combo vaccinated heifers compared to single vaccinated heifers at respective time points. AR1, circles represent individual animal cell counts and gray bars represent means ± SEM. NS, no significance. *p ≤ 0.05; **p ≤ 0.01; ***p ≤ 0.001.

To determine the biological significance of this observation *in vivo*, we also assessed CD4^+^ T cell proliferative responses in crossbred heifers co-vaccinated with RB51 and BCG. PBMCs isolated from combo vaccinated heifers were stimulated with the cognate antigen for each vaccine, γ-irradiated RB51 and PPDb, respectively. Comparison of RB51 (red circles) and combo vaccinated cattle (black circles) revealed no differences (p ≥ 0.092) between the RB51-specific CD4^+^ T cell-mediated proliferative responses in terms of magnitude or kinetics between vaccinate groups (**Figure 2A**). However, assessment of the PPDb-specific response between BCG (blue circles) and combo vaccinated cattle (black circles) showed statistical differences. The combo vaccinated cattle had higher numbers of proliferating CD4^+^ T cells in response to PPDb stimulation at 4-, 8-, 12-, 16-, and 20-weeks PV (p < 0.005, p < 0.02, p < 0.0001, p < 0.01, p < 0.002, respectively) when compared to BCG vaccinated cattle (**Figure 2B**). The combination also resulted in changes to the kinetics of the PPDb-specific proliferative response when compared to responses of BCG vaccinated cattle. Peak proliferation in combo vaccinated cattle was observed at 12-weeks PV in contrast to 8 weeks PV in BCG vaccinated cattle. Furthermore, the response was sustained longer in the combo vaccinates than BCG vaccinates, with statistical differences in proliferation still observed at 24 weeks PV (p < 0.01). Altogether, these data suggest that the addition of RB51 to BCG vaccination increased the magnitude and affects the kinetics of the BCG-specific responses, but the addition of BCG to RB51 has no effect on RB51-specific responses.

### 3.4 Magnitude of the Th1 functional potential of antigen-specific CD4^+^ T cells

**Figure 3:**
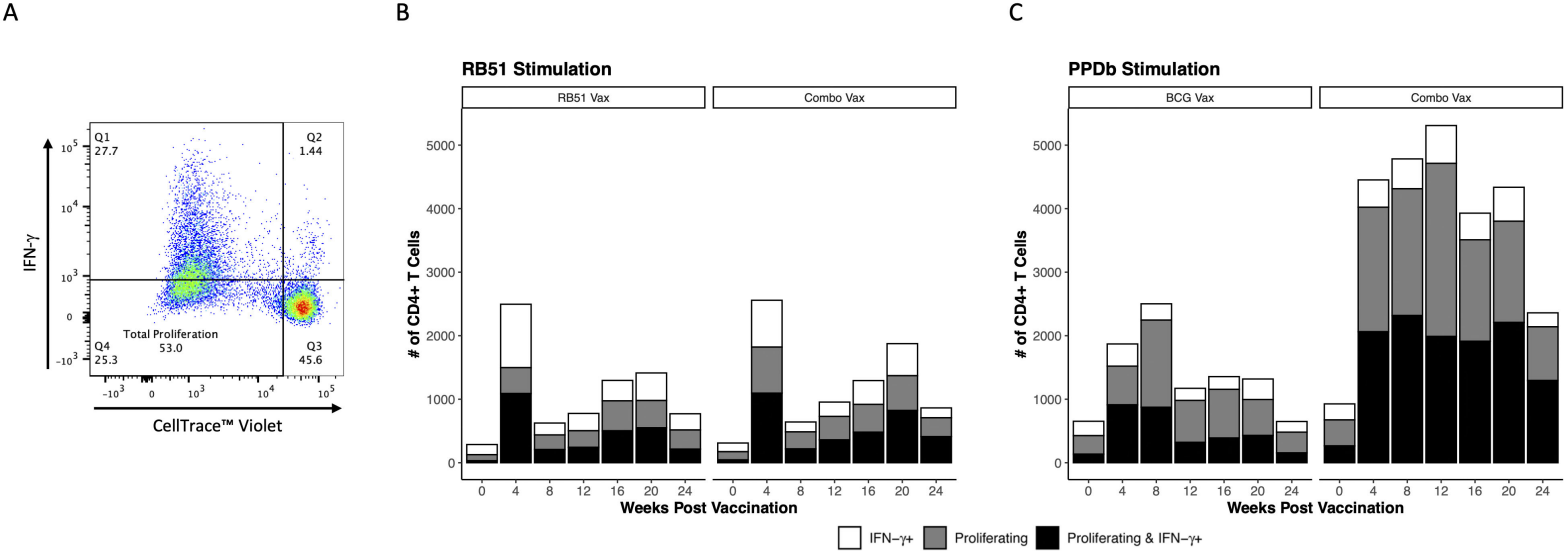
Th1 functional potential of vaccine-specific CD4^+^ T cells in dairy-beef heifers. **(A)** Quadrant gate defines functional subsets of CD4^+^ T cells: Q1, proliferating and IFN-γ^+^; Q2, IFN-γ^+^ only; Q3, unresponsive; Q4, proliferating only. **(B, C)** Bar height represents total average number of responding CD4^+^ T cells; broken down into IFN-γ^+^ only (white bars), proliferating only (gray bars), and proliferating and IFN-γ^+^ (black bars) subsets. **(B)** Mean number of RB51-specific CD4^+^ T cells exhibiting Th1 functionality in RB51 vaccinated (n = 8) and combo vaccinated (n = 8) heifers. **(C)** Mean number of BCG-specific CD4^+^ T cells exhibiting Th1 functional potential in BCG vaccinated (n = 8) and combo vaccinated (n = 8) heifers.

To further characterize the functional phenotype of the antigen-specific CD4^+^ T cell responses in all vaccine groups, we evaluated IFN-γ production as a measure of Th1-polarized effector function. CD4^+^ T cells were broken down into three functional groups, characterized by the response to antigen stimulation: proliferating and IFN-γ^+^ (**Figure 3A** Q2, **Figures 3B, C** white bars), IFN-γ^+^ only (**Figure 3A** Q2, **Figures 3B, C** white bars), and proliferating only (**Figure 3A** Q4, **Figures 3B, C** gray bars). When comparing the responses of functional groups between RB51 vaccinates and combo vaccinates following *in vitro* RB51 stimulation, we observed similar numbers of total responding cells at each time point and the functional group responses were consistent between the two vaccine groups across all time points analyzed (**Figure 3B**). However, in response to *in vitro* PPDb stimulation, combo vaccinated cattle had both higher numbers of total responding CD4^+^ T cells and higher numbers of CD4^+^ T cells in each of the three functional groups as compared to BCG vaccinated cattle at all time points PV (**Figure 3C**). The observed increase in the number of CD4^+^ T cells is predominately found within proliferating only and proliferating and IFN-γ^+^ populations. Consistent with previous findings, these data demonstrate that RB51 and BCG vaccination both result in Th1 polarized CD4^+^ T cell responses. We demonstrate that addition of BCG to the RB51 vaccine did not have any effect on the functional cell-mediated RB51 responses in cattle, but that combo vaccination did enhance numbers of PPDb-specific CD4^+^ T cells with a Th1-polarized effector response compared to BCG vaccination alone.

### 3.5 Repeatability of enhanced PPDb-specific CD4^+^ T cell proliferation

**Figure 4:**
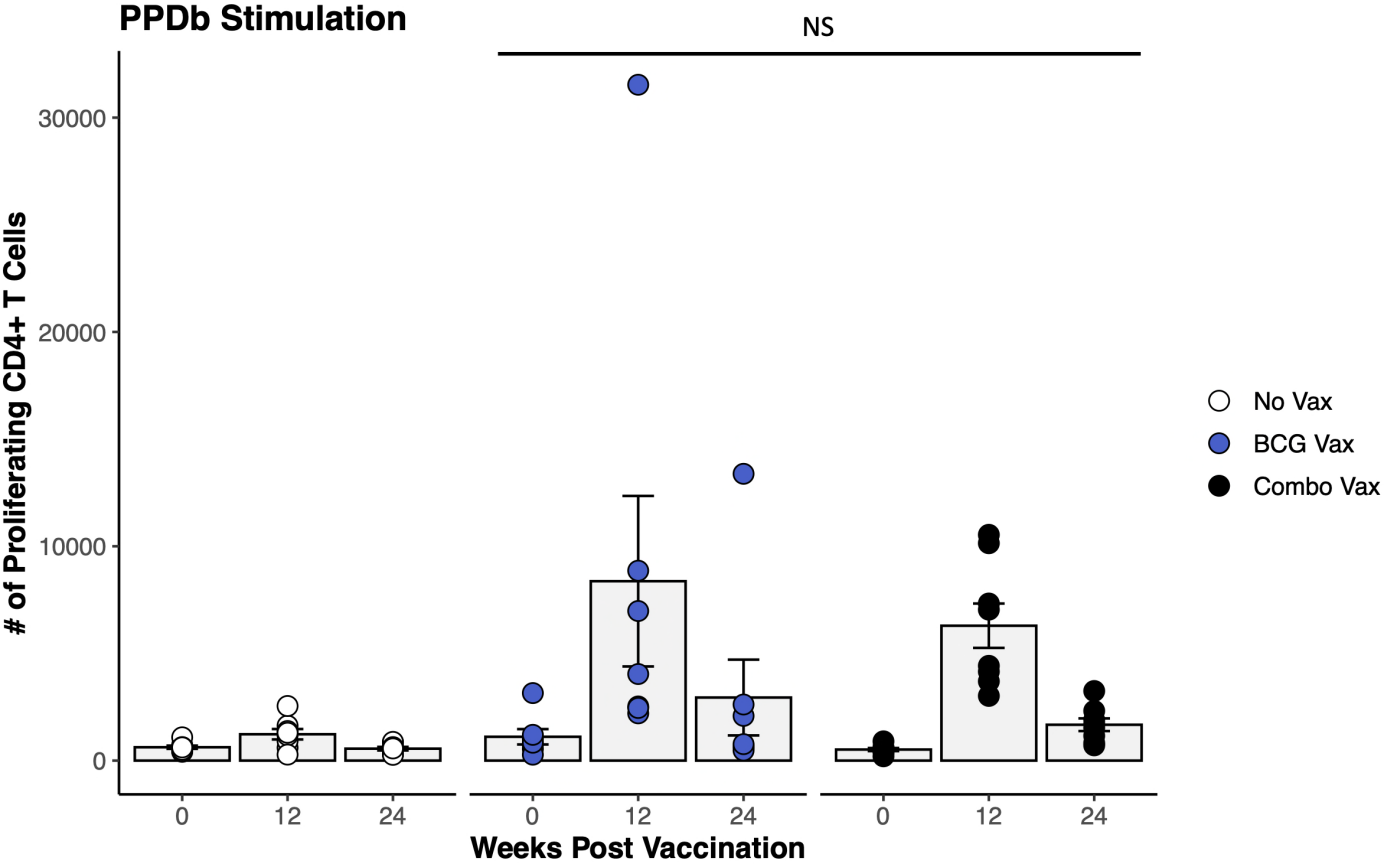
Proliferation of CD4^+^ T cells in response to *in vitro* PPDb stimulation in BCG and combo vaccinated Holstein steers. The average number of CD4^+^ T cells (gray bars) proliferating in response to PPDb stimulation from unvaccinated (white circles, n = 8), BCG vaccinated (blue circles, n = 7), and combo vaccinated (black circles, n = 8) Holstein steers. AR1, circles represent individual animal cell counts and gray bars represent means ± SEM. *NS, no significance*.

To assess this phenomenon of an enhanced BCG-specific CD4^+^ T cell response following combo vaccination of dairy-beef crossbred heifers with RB51 and BCG, a similar study was conducted in Holstein steers. Our analysis focused on evaluating proliferation at two time points prior to proposed *M. bovis* challenge, 12- and 24- weeks PV, as these represented the peak and contraction of the proliferative response, respectively. In Holstein steers, combo vaccination did not enhance the PPDb-specific CD4^+^ T cell proliferative responses when compared to BCG-only vaccinated animals. Proliferative responses were the same (p > 0.2) in the BCG vaccinates and the combo vaccinates at the time points analyzed (**Figure 4**). Analysis of the functional potential of the PPDb-specific CD4^+^ T cells showed similar numbers of responding cells and proportions between the three functional groups (**Supplementary Figure S2**). In contrast to the results reported for the dairy-beef crossbred heifers, the combo vaccination of Holstein steers with RB51 and BCG did not enhance BCG-specific CD4^+^ T cell responses.

## 4 Discussion

This study evaluated peripheral CD4^+^ T cell responses following vaccination of cattle with RB51 and BCG. We provide evidence that combo vaccination of dairy-beef crossbred cattle with RB51 and BCG enhanced the Th1-polarized cellular immune response to BCG. However, in a separate study using Holstein steers, we did not observe the increase in the BCG-specific CD4^+^ T cell response with combo vaccination.

We utilized a flow cytometry panel previously developed by our lab (44) and used an *in vitro* recall response assay to evaluate a time course of RB51-specific responses in cattle up to 24 weeks following vaccination. Our data demonstrated that CD4^+^ T cells proliferated significantly and produced IFN-γ in response to RB51 stimulation as early as 4 weeks PV. Further, we were able to define three subsets of RB51-specific functional CD4^+^ T cells: cells that only proliferated, cells that only produced IFN-γ, and cells that proliferated and produced IFN-γ. These results build on established findings following RB51 vaccination in cattle which demonstrated significant proliferative responses and IFN-γ production in PBMCs stimulated with γ-irradiated RB51 starting at 4 weeks post RB51 vaccination (45). Previously, proliferation was measured in the total PBMC population and cytokine production was evaluated with supernatant ELISAs, therefore the phenotype of proliferating cells and the source of the IFN-γ were not known. Dorneles et al. (16) described the phenotypic and functional characteristics of RB51-specific lymphocytes using flow cytometry to evaluate surface markers, proliferation, and IFN-γ production of PBMCs stimulated with *Brucella* antigens up to a year after RB51 vaccination. In that study, proliferating CD4^+^ and CD8^+^ T cells were observed as early as 4 weeks post RB51 vaccination, and the main source of IFN-γ was CD4^+^ T cells. However, proliferation and IFN-γ production were evaluated separately and therefore only casual conclusions could be made regarding the functional capacity of the RB51-specific CD4^+^ T cell populations.

In addition to RB51, we chose to stimulate PBMCs from RB51 vaccinated cattle with PPDb, the antigen used to evaluate *M. bovis*-specific cell-mediated responses. Unexpectedly, PPDb did not serve as a negative control and instead, proliferative responses in the CD4^+^ T cell population were observed starting at 8 weeks post-RB51 vaccination and continuing through 20 weeks PV. No significant proliferation occurred in PPDb-stimulated PBMCs prior to RB51 vaccination compared to unstimulated PBMCs (p = 0.58), suggesting that responses were due to RB51 vaccination. These observations led us to conclude that vaccination of cattle with RB51 was priming peripheral CD4^+^ T cells to respond to *M. bovis*-derived antigens *in vitro*. We had previously shown that PBMCs isolated from BCG vaccinated, RB51-naïve cattle did not proliferate or produce IFN-γ in response to RB51 stimulation (46). The unique one-directional *in vitro* priming effect of RB51 vaccination that we observed led us to explore whether we could exploit this response *in vivo* to enhance either vaccine-specific CD4^+^ T cell response by combo vaccinating cattle with RB51 and BCG.

We found that addition of BCG to the RB51 vaccine had no effect on proliferative CD4^+^ T cell responses to γ-irradiated RB51 stimulation. However, corresponding to the one-directional *in vitro* observations, we found that the addition of RB51 to the BCG vaccine significantly enhanced BCG-specific responses. Upon evaluation of the functional subsets of cells responding to antigen stimulations following RB51, BCG, or combo vaccination, we found that only the number of functional BCG-specific CD4^+^ T cells were enhanced following combo vaccination. The enhanced *in vitro* expansion of BCG-specific CD4^+^ T cells isolated from combo vaccinated heifers compared to BCG vaccinated heifers, whether IFN-γ^+^ or IFN-γ^−^, indicates the possibility of a more efficient *in vivo* cell-mediated immune response in the face of an *M. bovis* infection. Altogether, these results suggested that combo vaccination uniquely increased the BCG-specific Th1-polarized cell mediated immune responses but had no effect on the RB51-specific Th1 responses.

Since the animals in this study were not challenged with *M. bovis*, we can only speculate that the increased numbers of BCG-specific IFN-γ^+^ CD4^+^ T cells in combo vaccinated animals could contribute to enhanced protection against infection. There is evidence that the context in which IFN-γ is produced, not solely an increase in IFN-γ concentration or number of IFN-γ^+^ T cells, determines the outcome of *M. bovis* infections. Previous analysis of BCG-specific memory T cells suggests that IFN-γ^+^ central memory (T_CM_) are essential for protection against *M. bovis* (47, 48). Cytokine production by T_CM_ indicates that tumor necrosis factor α (TNF-α)^+^interleukin-2 (IL-2)^+^/IFN-γ^+^ polyfunctionality is associated with protection against *M. bovis*, while TNF-α^+^IFN-γ^+^ T_CM_ are negatively correlated with protection (49). Evaluation of systemic cytokine signatures suggests that IL-2 and IL-17A alone, and IL-1β and CXCL10 in conjunction with IFN-γ, are potential DIVA biomarkers and correlates of protection against *M. bovis* infection (50–54). Besides IFN-γ, the current study did not evaluate production of other Th1 cytokines, chemokines, surface markers, or other candidate biomarkers that may indicate protection against *M. bovis* infection. In future studies, we intend to more thoroughly evaluate BCG-specific cellular memory phenotypes, polyfunctionality, and the cytokine and chemokine environment in conjunction with *M. bovis* challenge.

Seeing that combo vaccination of dairy-beef cross heifers with RB51 and BCG enhanced BCG-specific cell-mediated immune responses, we repeated the study to compare BCG responses between single and combo vaccinates in a group of Holstein steers. In contrast to the original study, we did not observe differences in proliferative CD4^+^ T cell responses to PPDb between BCG vaccinated and combo vaccinated steers. Significant CD4^+^ proliferation to RB51 stimulation was observed in PBMCs isolated from combo vaccinated animals, indicating that the RB51 vaccine was administered to the combo vaccinate group (**Supplementary Figure S3**). Animals in the two vaccine studies were housed under the same conditions and the same route of vaccine administration was used, but cattle varied in age, sex, and genetic background and were housed in separate pastures. It has been established that stringent genetic selection for milk production in the Holstein breed over the past 60 years has affected many immune-related genes (55). Further, the genetic selection for milk production in Holsteins specifically influences PBMC transcriptional signatures in response to RB51 exposure (56). While age, sex, and environment of the animals used in the two studies along with assay variability may have been minor confounding variables, we hypothesize that differences in host genetic background led to the variation in the T cell response to combo vaccination.

Our results indicate that combo vaccinating cattle with RB51and BCG may be an option to enhance immune responses to the BCG vaccination. Administration of RB51 with BCG significantly enhanced *in vitro* proliferative CD4^+^ T cell responses to mycobacterial antigens compared to vaccination with only BCG in dairy-beef crossbred heifers, however, this observation was not replicated in Holstein steers. While batch effects and/or environmental differences are possible factors that could affect results, further investigation into the possible effect of genetic background on immune responses to BCG and RB51 administered alone or together, is currently ongoing. Assessment of how the enhanced T cell response observed in this study correlates to protection against challenge with both *B. abortus* and *M. bovis* is also of interest. The effect of cattle breed, age, and sex on immune responses should be evaluated further to describe the full effect of RB51 and BCG combo vaccination across the bovine species. Notably, the evaluation of combo vaccination during disease challenge should be assessed to fully consider the potential application of altered vaccination strategies for beef and dairy cattle producers.

## Data Availability

The datasets presented in this study can be found in online repositories. The names of the repository/repositories and accession number(s) can be found below: Ag Data Commons https://doi.org/10.15482/USDA.ADC/26871160.v1.
