# Supplementary material for: Effect of co-vaccination of cattle with RB51 and BCG on vaccine-specific CD4+ T cell responses
Source: Front Immunol. 2025 Aug 27;16:1664398. doi: 10.3389/fimmu.2025.1664398 (PMC12420227; doi:10.3389/fimmu.2025.1664398)
Supplement: Supplementary file 1 [file DataSheet1.docx]

Supplementary Material

# Supplementary Figures

**
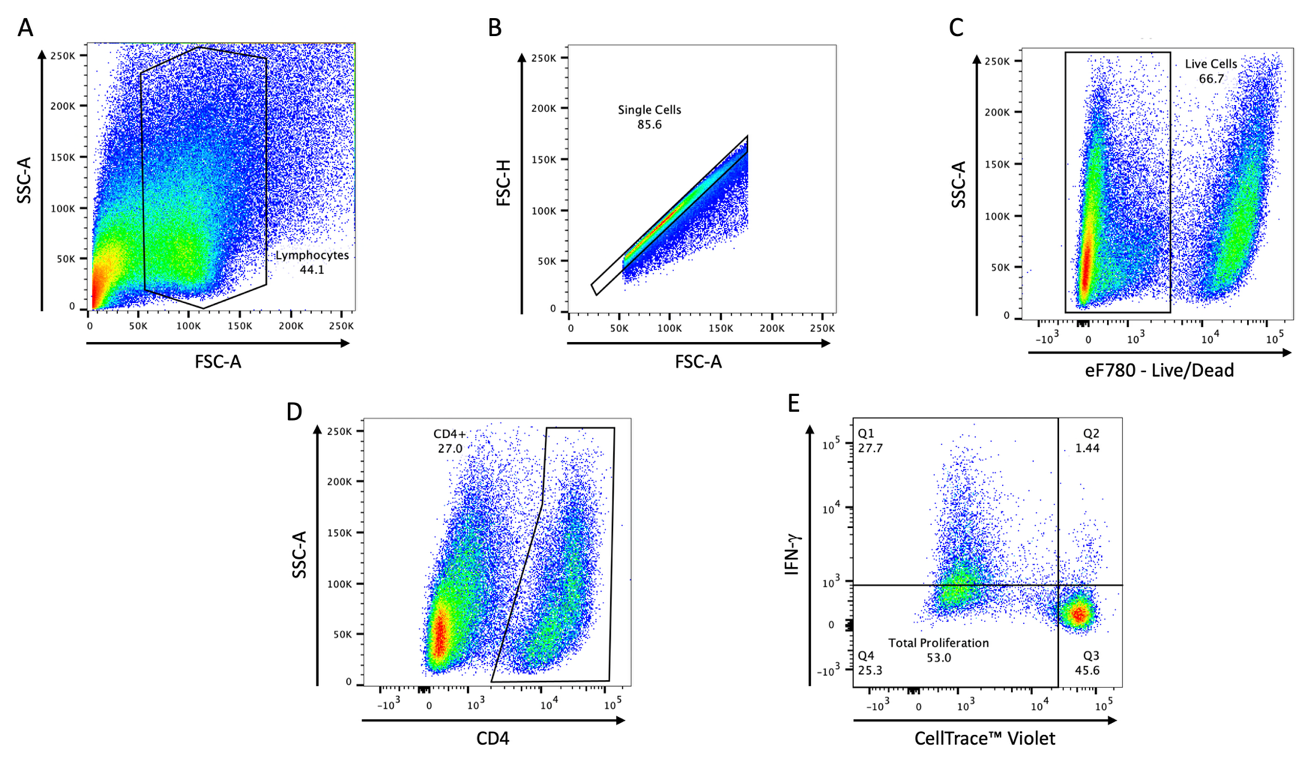
**

**Supplementary Figure 1.** Gating strategy used for flow cytometry analysis. Representative dot plots are shown describing the gating strategy for **(A)** lymphocytes, **(B)** single cells, **(C)** live cells, **(D)** CD4^+^ T population, **(E)** antigen-specific proliferation and/or IFN-γ production.


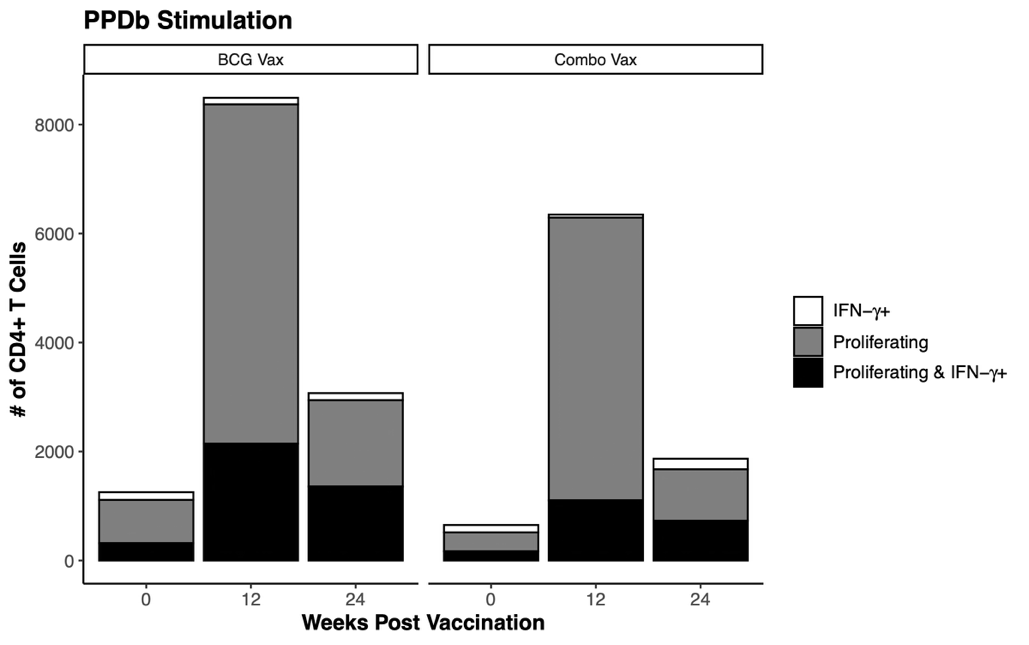


**Supplementary Figure 2.** Th1 functional potential of *Mycobacterium*-specific CD4^+^ T cells from Holstein steers. Bar height represents total average number of responding CD4^+^ T cells; broken down into IFN-γ^+^ only (white bars), proliferating only (gray bars), and proliferating and IFN-γ^+^ (black bars) subsets.


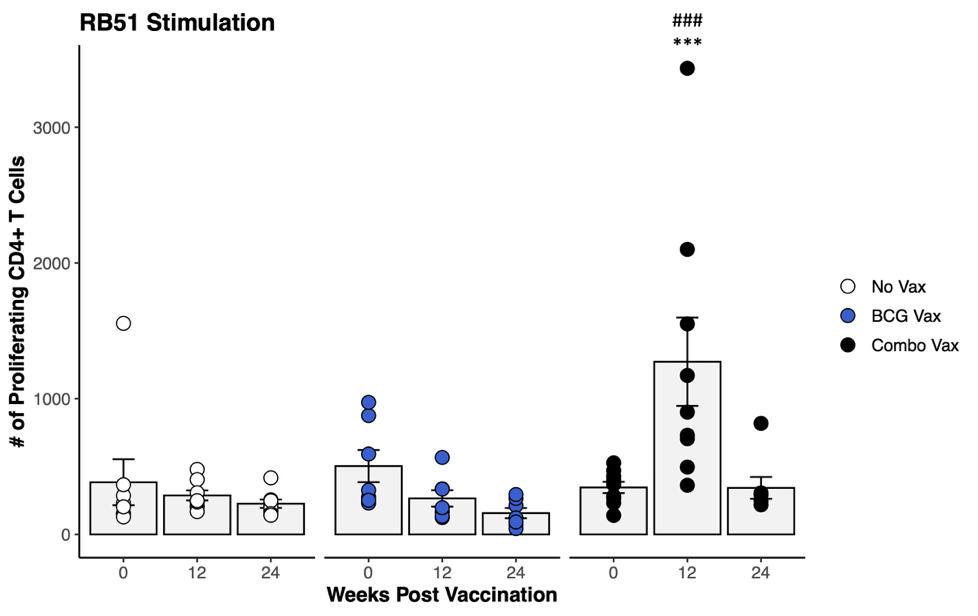


**Supplementary Figure 3.** Proliferative CD4^+^ T cell response to γ-irradiated RB51 stimulation in Holstein steers. Mean numbers of RB51-specific CD4^+^ T cells (gray bars) in unvaccinated (white circles), BCG vaccinated (blue circles), and combo vaccinated steers (black circles). *, combo vaccinated compared to unvaccinated steers. #, combo vaccinated compared to BCG vaccinated steers. ****, ### p ≤ 0.001.*
